# Geographical origin of *Leucobryum boninense* Sull. & Lesq. (Leucobryaceae, Musci) endemic to the Bonin Islands, Japan

**DOI:** 10.1002/ece3.492

**Published:** 2013-02-15

**Authors:** Emiko Oguri, Tomio Yamaguchi, Hiromi Tsubota, Hironori Deguchi, Noriaki Murakami

**Affiliations:** 1 Makino Herbarium, Graduate School of Science and Engineering, Tokyo Metropolitan University1-1 Minami-osawa, Hachioji, Tokyo, 192-0397, Japan; 2 Department of Biological Science, Graduate School of Science, Hiroshima University1-3-1 Kagamiyama, Higashi-hiroshima, Hiroshima, 739-8526, Japan; 3 Miyajima Natural Botanical Garden, Graduate School of Science, Hiroshima University1156-2 Mitsumaruko-yama, Miyajima-cho, Hatsukaichi, Hiroshima, 739-0543, Japan

**Keywords:** Bonin Islands, bryophytes, chloroplast DNA sequences, *Leucobryum*, *Leucobryum boninense*, *matK*, molecular phylogeny, Musci, oceanic island

## Abstract

*Leucobryum boninense* is endemic to the Bonin Islands, Japan, and its related species are widely distributed in Asia and the Pacific. We aimed to clarify the phylogenetic relationships among *Leucobryum* species and infer the origin of *L. boninense*. We also describe the utility of the chloroplast *trnK* intron including *matK* for resolving the phylogenetic relationships among *Leucobryum* species, as phylogenetic analyses using *trnK* intron and/or *matK* have not been performed well in bryophytes to date. Fifty samples containing 15 species of *Leucobryum* from Asia and the Pacific were examined for six chloroplast DNA regions including *rbcL*, *rps4*, partial 5′ *trnK* intron, *matK*, partial 3′ *trnK* intron, and *trnL*-*F* intergenic spacer plus one nuclear DNA region including ITS. A molecular phylogenetic tree showed that *L. boninense* made a clade with *L. scabrum* from Japan, Taiwan and, Hong Kong; *L. javense* which is widely distributed in East and Southeast Asia, and *L. pachyphyllum* and *L. seemannii* restricted to the Hawaii Islands, as well as with *L. scaberulum* from the Ryukyus, Japan, Taiwan, and southeastern China. *Leucobryum boninense* from various islands of the Bonin Islands made a monophylic group that was closely related to *L. scabrum* and *L. javense* from Japan. Therefore, *L*. *boninense* may have evolved from *L. scabrum* from Japan, Taiwan, or Hong Kong, or *L. javense* from Japan. We also described the utility of *trnK* intron including *matK*. A percentage of the parsimony-informative characters in *trnK* intron sequence data (5.8%) was significantly higher than that from other chloroplast regions, *rbcL* (2.4%) and *rps4* (3.2%) sequence data. Nucleotide sequence data of the *trnK* intron including *matK* are more informative than other chloroplast DNA regions for identifying the phylogenetic relationships among *Leucobryum* species.

## Introduction

Bryophyte species tend to have broad geographical distribution with a morphological uniformity in comparison with those of seed plants. In the Northern Hemisphere, more than 60% of the flora of the Arctic and boreal regions is made up of the same species (Schofield and Crum 1972). A single sporangium of a bryophyte may contain thousands and sometimes over 50 million spores that have the capacity for long-distance dispersal over thousands of kilometers (Kreulen 1972; van Zanten 1978). Producing abundant air-borne diaspores would appear to guarantee a wide distribution of many bryophyte species (Schofield and Crum 1972). In contrast, extreme geographical isolation such as on oceanic islands affects diversification and speciation, even though bryophyte species have the capability for long-distance dispersal (Oguri et al. 2008). Therefore, oceanic islands may provide models for research on patterns and processes of bryophyte evolution and speciation.

The Bonin (Ogasawara) Islands are oceanic islands located in the northwestern Pacific Ocean, approximately 1000 km south of Tokyo, Japan (Asami 1970). These islands were formed during the Paleocene and rose above sea level before the middle Pleistocene (Kaizuka 1977; Imaizumi and Tamura 1984). Approximately 300 indigenous species of vascular plants are known from these islands, and their percentage of endemism is estimated to be as high as 40–43% (Kobayashi 1978; Ono et al. 1986). A total of 155 species of bryophytes (48 genera and 81 species of mosses, 33 genera and 74 species of liverworts and hornworts) are currently known from the Bonin Islands (Inoue and Iwatsuki 1969, 1970, 1984; Inoue 1970a,b; Iwatsuki 1985; Furuki et al. 1991). The percentage of bryophyte endemism is approximately 5%, which is much lower than that of vascular plants.

Among bryophyte taxa growing on the Bonin Islands, members of the genus *Leucobryum* Hampe (Leucobryaceae, Musci) have been taxonomically well studied by Yamaguchi (1993) and Oguri et al. (2008). This genus is one of the most widely distributed moss genera, containing several widespread species. According to van der Wijk et al. (1964), it includes approximately 180 species. Among members of *Leucobryum*, *L. juniperoideum* (Brid.) Müll.Hal. is widely distributed in Asia, Europe, Macaronesia, and Madagascar, whereas *L. glaucum* (Hedw.) Ångstr. is widely distributed throughout temperate to cool temperate regions in the Northern Hemisphere (Yamaguchi 1993; Vanderpoorten et al. 2003). In contrast, some endemic species are observed on oceanic islands such as the Hawaiian Islands and the Bonin Islands. *Leucobryum pachyphyllum* Müll.Hal. and *L. seemannii* Mitt. are endemic to the Hawaii Islands (Bartram 1933; Staples et al. 2004), whereas *L. boninense* Sull & Lesq. (Oguri et al. 2008) is restricted to the Bonin Islands.

**Figure 1:**
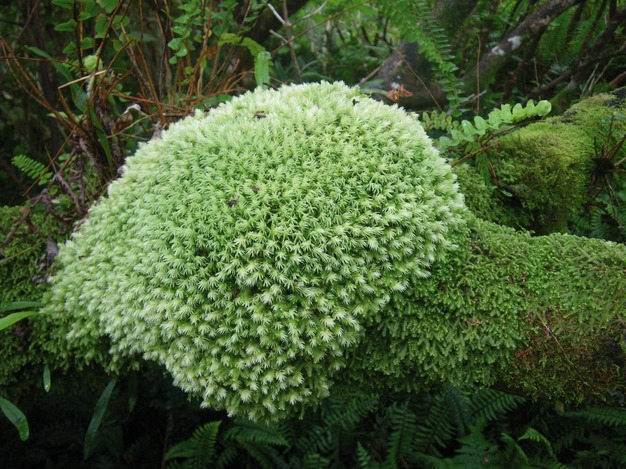
*Leucobryum boninense* Sull. & Lesq. growing on Kita-iwo Island.

*Leucobryum boninense* is characterized by its perichaetia terminal on short lateral branches and papillose proration on the abaxial surface of apical parts of leaves (Fig. 1; Yamaguchi 1993). This species seems to be closely related to *L. scaberulum* Cardot based on morphological characters. In fact, *L. scaberulum* was treated as a synonym of *L. boninense* by Yamaguchi (1993).

Molecular phylogenetic analyses of the genus *Leucobryum* have been performed based on sequence data of internal transcribed spacer (ITS) regions of ribosomal DNA and chloroplast *rbcL* gene. The results showed that the endemic species, *L. boninense*, is closely related to *L. scaberulum*, *L. scabrum* Sande Lac., and *L. javense* (Brid.) Mitt. (Oguri et al. 2003, 2008). All three species are widely distributed; *L. javense* is widely distributed in East and Southeast Asia, and *L. scabrum* and *L. scaberulum* occur in East Asia (Yamaguchi 1993). Nevertheless, two previous molecular phylogenetic studies did not include plant samples from various parts of the distribution areas and were performed based only on ITS and *rbcL* DNA sequence regions. Therefore, detailed phylogenetic relationships among *L. boninense* and its related species remain poorly understood. *matK*, encoding a splicing-associated maturase in the land plant chloroplast genome, is a very popular region for phylogenetic study and has been extensively applied to reconstruct angiosperm phylogeny (Rev. Müller et al. 2006). However, the utility of *matK* in bryophyte phylogeny is largely unknown. Only one molecular phylogenetic study has been conducted by Long et al. (2000), but it was based on partial *matK* sequence data.

In this study, we collected *L. boninense* samples and those of its related taxa from various parts of their distribution and performed molecular phylogenetic studies to clarify the phylogenetic relationships among *Leucobryum* species and to infer the origin of *L. boninense*, which is restricted to the Bonin Islands. Phylogenetic trees were constructed based on the combined nucleotide sequences of *rbcL*, *rps4*, 5′ *trnK* intron, *matK*, 3′ *trnK* intron, *trnL*-*F* intergenic spacer, and ITS regions. Moreover, we verified amplification of the *matK* region for six moss species, in addition to the *Leucobryum* species and obtained their sequence data using six primers including four new internal primers designed in this study.

## Materials and methods

### Plant materials

Fifty samples belonging to 15 species of *Leucobryum* were collected from Asia and the Pacific regions (Table 1). *Leucobryum sanctum* (Brid.) Hampe was used as an outgroup for the phylogenetic analysis, based on a previous molecular phylogenetic study of the entire genus by Oguri et al. (2003). Six additional moss species of different genera were also included in our analyses to conduct polymerase chain reaction (PCR) amplification of *trnK* intron including *matK* and to obtain their sequence data: *Tetraphis pellucida* Hedw. (Tetraphidaceae), *Brothera leana* (Sull.) Müll.Hal. (Dicranaceae), *Dicranodontium denudatum* (Brid.) E.G.Britt. ex Williams (Dicranaceae), *Hypnum plumaeforme* Wilson (Hypnaceae), *Isopterygium propaguliferum* Toyama (Hypnaceae), and *Rhytidium rugosum* (Hedw.) Kindlb. (Hylocomiaceae) (Appendix S1). Voucher specimens are deposited at Herbarium of Hiroshima University, Hiroshima, Japan (HIRO) or Makino Herbarium (MAK), Tokyo Metropolitan University, Tokyo, Japan.

**Table 1:** List of taxa investigated in this study, voucher specimen, origin of sample, and accession numbers

Taxon		Voucher specimen	Origin of sample	*rbcL*	*rps4*	*trnK* intron	*trnL-F*	ITS
*Leucobryum aduncum Dozy & Molk*.	1	HIRO 140862	Indonesia. Borneo	AB124781*	AB740043	AB742458	AB742374	AB125287*
	2	HIRO 140934	Indonesia. Borneo	AB739623	AB740044	AB742459	AB742375	AB763349
	3	HIRO 138507	Malaysia. Malay Pen.	AB739624	AB740045	AB742460	AB742376	AB763350
	4	HIRO 166266	Sri Lanka. Nuara Eliya Dist.	AB739625	AB740046	AB742461	AB742377	AB763351
	5	HIRO 166267	Sri Lanka. Nuara Eliya Dist	AB739626	AB740047	AB742462	AB742378	AB763352
	6	HIRO 166239	Vanuatu	AB739627	AB740048	AB742463	AB742379	AB763353
*L. albidum* (P.Beauv.) Lindb.		HIRO 166241	U. S. A. Florida	AB124784*	AB740049	AB742464	AB742380	AB125288*
*L. boninense* Sull. & Lesq.	1	MAK B119207	Japan. Ogasawara Isls. Chichijima Isl.	AB739629	AB740050	AB742465	AB742381	AB763354
	2	MAK B119201	Japan. Ogasawara Isls. Hahajima lsl.	AB739630	AB740051	AB742466	AB742382	AB763355
	3	MAK B119184	Japan. Ogasawara Isls. Anijima lsl.	AB739631	AB740052	AB742467	AB742383	AB763356
	4	MAK B119190	Japan. Ogasawara Isls. Anijima lsl.	AB739632	AB740053	AB742468	AB742384	AB763357
	5	MAK B119192	Japan. Ogasawara Isls. Anijima lsl.	AB739633	AB740054	AB742469	AB742385	AB763358
	6	HIRO 268806	Japan. Ogasawara Isls. Kita-iwo Isl.	AB739634	AB740055	AB742470	AB742386	AB763359
	7	HIRO 269656	Japan. Ogasawara Isls. Kita-iwo Isl.	AB739635	AB740056	AB742471	AB742387	AB763360
*L. bowringii* Mitt.		HIRO 139187	Japan. Yakushima Isl.	AB124790*	AB740057	AB742472	AB742388	AB125290*
*L. candidum* (Brid. ex P.Beauv.)		HIRO 203728	New Zealand	AB288196**	AB740058	AB742473	AB742389	AB285170**
*L. chlorophyllosum* Müll.Hal.	1	HIRO 140710	Indonesia. Borneo	AB124792*	AB740059	AB742474	AB742390	AB125291*
	2	HIRO 140820	Indonesia. Borneo	AB739636	AB740060	AB742475	AB742391	AB763361
	3	MAK B119208	Philippines	AB739637	AB740061	AB742476	AB742392	AB763362
*L. glaucum* (Hedw.) Ångstr.		HIRO 138407	Japan. Hokkaido	AB124788*	AB740062	AB742477	AB742393	AB125292*
*L. javense* (Brid.) Mitt.	1	HIRO 120786	Japan. Amami-oshima Isl.	AB739638	AB740063	AB742507	AB742394	AB194567
	2	MAKB119211	Japan. Amami-oshima Isl.	AB739639	AB740064	AB742479	AB742395	AB763363
	3	HIRO 120264	Taiwan. Pingtung County	AB124791*	AB740065	AB742480	AB742396	AB125294*
	4	HIRO 138505	Malaysia. Malay Pen.	AB739640	AB740066	AB742481	AB742397	AB763364
	5	HIRO 138508	Malaysia. Malay Pen.	AB739641	AB740067	AB742482	AB742398	AB763365
	6	HIRO 166240	Thailand. Doi Inthanon	AB739642	AB740068	AB742483	AB742399	AB763366
	7	HIRO 166247	Malaysia. Borneo	AB739643	AB740069	AB742484	AB742400	AB763367
*L. juniperoideum* (Brid.) Müll.Hal.		HIRO 139224	Japan. Yakushima Isl.	AB124786*	AB740070	AB742485	AB742401	AB125295*
*L. pachyphyllum* Müll.Hal.		HIRO 119467	Hawaii. Oahu Isl.	AB124782*	AB740071	AB742486	AB742402	AB125296*
*L. sanctum* (Brid.) Hampe		HIRO 140948	Indonesia. Borneo	AB124787*	AB740072	AB742487	AB742403	AB125297*
*L. scaberulum* Cardot	1	HIRO 136706	Hong Kong. Lantau Isl.	AB288199**	AB740073	AB742488	AB742404	AB285178**
	2	HIRO 136707	Hong Kong. New Territories	AB739644	AB740074	AB742489	AB742405	AB285179**
	3	MAK B119196	Hong Kong. New Territories	AB739645	AB740075	AB742490	AB742406	AB763368
	4	MAK B119194	China. Guandong Province	AB739646	AB740076	AB742491	AB742407	AB763369
	5	HIRO 134131	Japan. Iriomote lsl.	AB739647	AB740077	AB742492	AB742408	AB285173**
	6	HIRO 120155	Taiwan. Taichung County	AB739648	AB740078	AB742493	AB742409	AB285174**
	7	HIRO 120368	Taiwan. Nantou County	AB739651	AB740081	AB742496	AB742412	AB285175**
	8	HIRO 148838	Taiwan. Ilan Hsien/Taipei Hsien	AB288198**	AB740082	AB742497	AB742413	AB285176**
	9	HIRO 148840	Taiwan. Ilan Hsien/Taipei Hsien	AB739652	AB740083	AB742498	AB742414	AB285177**
*L. scabrum* Sande Lac.	1	MAK B119193	Japan. Wakayama-ken	AB739653	AB740084	AB742499	AB742415	AB763371
	2	HIRO 139186	Japan. Yakushima Isl.	AB124793*	AB740085	AB742500	AB742416	AB125298*
	3	MAK B119212	Japan. Amami-oshima Isl.	AB739654	AB740086	AB742501	AB742417	AB763372
	4	MAK B119210	Japan. Amami-oshima Isl.	AB739655	AB740087	AB742502	AB742418	AB763373
	5	HIRO 218554	Japan. Okinawa Isl.	AB739656	AB740088	AB742503	AB742419	AB763374
	6	HIRO 120226	Taiwan. Pingtung County	AB739657	AB740089	AB742504	AB742420	AB763375
	7	HIRO 120156	Taiwan. Taichung County	AB739649	AB740079	AB742494	AB742410	AB285180**
	8	HIRO 120158	Taiwan. Taichung County	AB739650	AB740080	AB742495	AB742411	AB763370
	9	HIRO 136709	Hong Kong. New Territories	AB739658	AB740090	AB742505	AB742421	AB763376
*L seemannii* Mitt.		HIRO 119505	Hawaii. Maui Isl.	AB739659	AB740091	AB742508	AB742422	AB285183**
*L. sumatranum* (Brid.) Hampe ex M.Fleisch.		HIRO 166243	Malaysia. Borneo	AB124785*	AB740092	AB742506	AB742423	AB125299*
								

* Oguri et al. 2003

** Oguri et al. 2008

### DNA extraction, PCR amplification, and sequencing

Total DNA was extracted either from fresh samples or dried herbarium specimens using the phenol-chloroform method of Tsubota et al. (2002) with some modifications. Six cpDNA regions, *rbcL*, *rps4*, 5′ *trnK* intron, *matK*, 3′ *trnK* intron, and *trnL*-*F* intergenic spacer and one nrDNA region, ITS were amplified by PCR using a thermal cycler (Table 2). Each fragment was amplified with PrimeSTAR Max DNA Polymerase (TaKaRa Bio, Otsu, Shiga, Japan) using 10 μl reactions volumes in a thermal cycle with the following conditions: 98°C for 30 sec followed by 30 cycles of 98°C for 10 sec, 55°C for 5 sec, 72°C for 30 sec and 72°C for 30 sec. After confirming PCR amplification on a 1.0% agarose gel, the amplified products were incubated at 37°C for 30 min and 80°C for 20 min with ExoSAP-IT (usb, Cleveland, OH, USA) to remove any excess primers and nucleotides. Eight primers for *rbcL*, two primers for *rps4*, six primers for *trnK* intron including *matK*, two primers for *trnL-F*, and five primers for ITS were used for the cycle sequencing reactions (Table 2) with an ABI PRISM BigDye Terminator Cycle Sequencing Kit v.3.1 (Applied Biosystems, Foster City, CA, USA). The sequencing reaction products were purified, concentrated by ethanol precipitation with sodium acetate and their nucleotide sequences were determined using an automated DNA sequencer (ABI PRISM 3100, Applied Biosystems). The obtained sequences were submitted to the DDBJ database (Table 1 and Appendix S1).

**Table 2:** PCR primers used in this study

Analyzed region	Primer name	Sequence	References
*rbcL*	**atbB175R**	TGT TGA ACT TCA CAA GTA ACA	Manhart 1994
	*rbcL* 256	GCT ATG ATC TTG AAG CAG TTC CTG GAG AAG	Tsubota et al. 2000
	*rbcL* 549	TGT CTT CGT GGT GGA C	Tsubota et al. 1999
	*rbcL* 919G	CAT GGT ATG CAT TTC CGT GTA	Tsubota et al. 2001
	*rbcL* 600R	GTG AAA TCA AGT CCA CCA CG	Tsubota et al. 1999
	*rbcL* 1098R	AAC ACC TGG TAA AGA AAC C	Tsubota et al. 1999
	*rbcL* 1346hR	GCA GCT AAT TCA GGA CTC C	Tsubota et al. 1999
	***trnRn***	GGG TTA GAA GGG ATT CGA ACC CTT GAC	Tsubota et al. 1999
*rps4*	***rps5***	ATG TCC CGT TAT CGA GGA CCT	Nadot et al. 1994
	***trnS***	TAC CGA GGG TTC GAA TC	Souza-Chies et al. 1997
*trnK* intron	***trnK* [tRNA-Lys(UAA)exonl]**	CCG ACT AGT TCC GGGTTCGA	Demesure et al. 1995
(including *matK)*	*trnK* aF	ARW TTC ATC CAA ACC ATT GAC AAG G	Designed this study
	*matK* 410F	TAT CAA TCT ATT CAT TCY GTA TTT CCT TTT	Designed this study
	*matK* 410R	AAA AGG AAA TAC RGA ATG AAT AGA TTG ATA	Designed this study
	*trnK* aR	ATT GCA CAC GGC TTT CTC TAT GT	Designed this study
	***trnK* [tRNA-Lys(UAA)exon2]**	CAA CGG TAG AGT ACT CGG CTT TTA	Demesure et al. 1995
*trnL-F*	**c**	CGA AAT CGG TAG ACG CTA CG	Taberlet et al. 1991
	**f**	ATT TGA ACT GGT GAC ACG AG	Taberlet et al. 1991
ITS	**18S1659B**	CGT CGC TCC TAC CGA TTG	Oguri et al. 2003
	18S1764B	AGA GGA AGG AGA AGT CGT AAC	Oguri et al. 2003
	5.8S10B	CTC AGC AAC GGA TAT CTT GG	Oguri et al. 2003
	26S102BR	CCG GTT CGC TCG CCG	Oguri et al. 2003
	**26S166BR**	GAG GAC GCT TCT CCA GAC TAC	Oguri et al. 2003

PCR amplification primers are shown in bold.

### Phylogenetic analysis

We obtained *rbcL* sequence data of 14 samples belonging to 13 taxa and ITS sequence data of 21 samples belonging to 14 taxa of the genus *Leucobryum* from the DNA database. The obtained *rbcL*, *rps4*, 5′ *trnK* intron, *matK*, 3′ *trnK* intron, *trnL*-*F*, and ITS sequences were separately aligned using the program MUSCLE (Edgar 2004).

We performed the Incongruence Length Difference (ILD) test (Farris et al. 1995) implemented in PAUP* version 4.0 beta (Swofford 2002) before phylogenetic reconstruction to confirm topological congruence between each DNA region. One hundred partition homogeneity replicates were implemented in the test using the heuristic search option with 100 random addition sequences. And then, we performed molecular phylogenetic analyses with combined all six chloroplast DNA plus one nuclear DNA sequences. When these analyses were carried out, identical sequences were pruned to include only one representative from each species. Therefore, a total of 35 operational taxonomic units, including outgroup, were used for the following analyses.

Bayesian inference (BI) analysis was performed using MrBayes version 3.1.2 (Ronquist and Huelsenbeck 2003). The best-fitting model for nucleotide substitution was selected for the combined seven regions based on Akaike information criterion (Akaike 1974) implemented in MrModeltest 2.2 (Nylander 2004), and GTR +I + G model was chosen. The analysis was performed for 1,000,000 generations with four chains, with samples taken every 100 generations.

Maximum likelihood (ML) analysis was conducted with PAUP* 4.0b10 using the best-fitting model GTR + I + G chosen by MrModeltest 2.2. A heuristic search algorithm was engaged with 100 random addition replicates and tree-bisection-reconnection (TBR) branch-swapping, and MulTrees on. The ML bootstrap value were computed in PAUP* 4.0b10 by running 1000 replicates with a full heuristic search using 100 random addition sequences, TBR branch-swapping, and MulTrees off (holding one tree at each step).

Maximum parsimony (MP) analysis was performed using PAUP* 4.0b10. A heuristic search algorithm was engaged with 100 random addition replicates and TBR branch-swapping, and MulTrees on. Parsimony bootstrap values were calculated using PAUP* 4.0b10. The bootstrap analysis used 1,000 bootstrap replicates, the heuristic search algorithm, 100 random addition sequences, TBR branch-swapping, and MulTrees off (holding one tree at each step).

## Results

### Sequence characteristics

Table 3 summarizes the sequence information for all *rbcL*, *rps4*, partial 5′ *trnK* intron, *matK*, partial 3′ *trnK* intron, *trnL-F*, and ITS regions, including the length of each region, numbers of variable and parsimony-informative sites, number of most parsimonious trees, tree length, consistency index (CI), and retention index (RI).

**Table 3:** Phylogenetic features of obtained nucleotide sequences of cpDNA and nrDNA in this study

			*trnK* intron				
					
	*rbcL*	*rps4*	5′ *trnK* intron	*matK*	3′ *trnK* intron	*trnK* intron	*trnL-F*
Aligned length (bp)	1428	471	322	1524	136	1982	439
bp included in analyses	1428	467	316	1521	132	1969	429
Variable characters	52 (3.6%)	24 (5.1%)	33 (10.4%)	129 (8.5%)	15 (11.4%)	177 (9.0%)	37 (8.6%)
Parsimony-informative chars.	34 (2.4%)	15 (3.2%)	21 (6.6%)	87 (5.7%)	7 (5.3%)	115 (5.8%)	26 (6.1%)
Number of trees (MP)	1	4	1	960	1	318	37
Tree length	69	28	38	164	17	221	45
CI	0.783	0.857	0.947	0.787	1.000	0.824	0.844
RI	0.885	0.913	0.971	0.904	1.000	0.916	0.936

	ITS	Combined
Aligned length (bp)	920	5240
bp included in analyses	589	4882
Variable characters	241 (40.9%)	730 (15.0%)
Parsimony-informative chars.	151 (25.6%)	531 (10.9%)
Number of trees (MP)	1	2
Tree length	392	1120
CI	0.778	0.768
RI	0.894	0.862

CI = Consistency index; RI = Retention index.

The ILD test did not detect incongruence between each pair of DNA data sets tested (combined data of the seven regions: *rbcL* + *rps4* + 5′ *trnK* intron + *matK* + 3′ *trnK* intron + *trnL-F* + ITS, *P =* 0.01; other data not shown). Based on these results, we combined all seven DNA sequences into one large data set, and the obtained phylogenetic results based on the combined data are shown (Table 3). The total aligned length for the combined sequences was 5,240 characters and 531 (10.9%) characters were parsimony informative. Parsimony analysis of all seven data regions resulted in two MP trees (Tree length = 1120, CI = 0.768, RI = 0.862).

We also tested the utility of *matK* for resolving phylogenetic relationships among *Leucobryum* species. The total aligned length for *trnK* intron including *matK* was 1,969 characters and 115 (5.8%) characters were parsimony informative. A percentage of parsimony-informative characters of *trnK* intron sequence data was significantly higher than that of other chloroplast sequence data (*rbcL*: 34 characters, 2.4%; *rps4*: 15 characters, 3.2%), except for *trnL-F* sequence data (26 characters, 6.1%).

We sequenced the chloroplast *trnK* intron including *matK* from six additional moss species of other genera including *Tetraphis pellucida* (Tetraphidaceae), *Brothera leana* (Dicranaceae), *Dicranodontium denudatum* (Dicranaceae), *Hypnum plumaeforme* (Hypnaceae), *Isopterygium propaguliferum* (Hypnaceae), and *Rhytidium rugosum* (Hylocomiaceae) (Appendix S1). The region was not amplified for the Hepaticae and Anthocerotae plant materials when we used PCR primers for exon 1 and exon 2 of the *trnK* intron (Demesure et al. 1995; see also Table 2).

### Phylogenetic analyses

**Figure 2:**
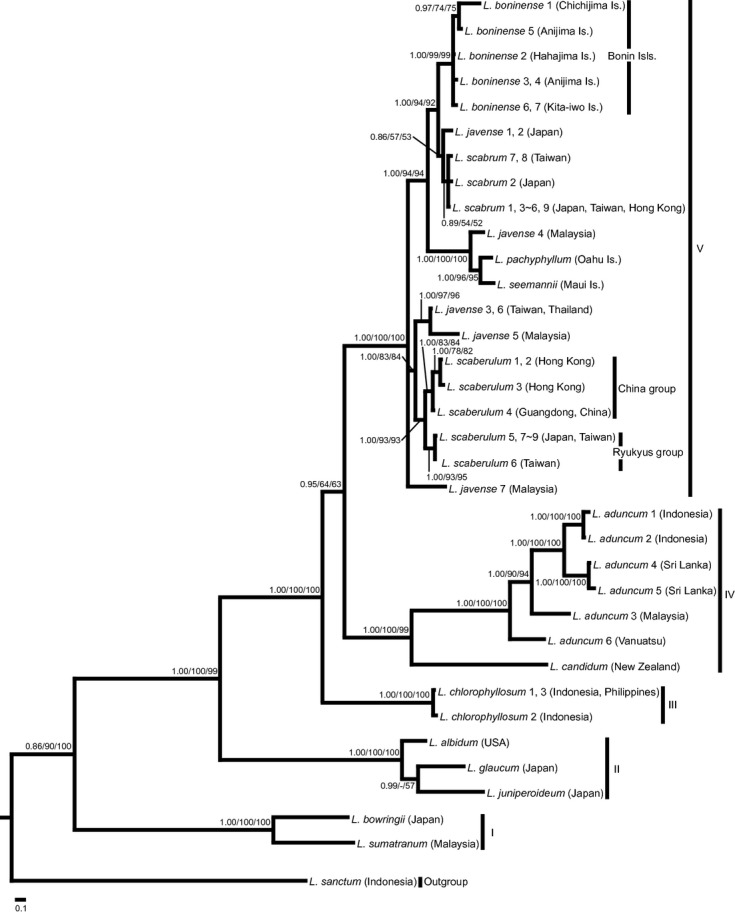
Molecular phylogenetic tree of *Leucobryum* species inferred from combined sequence data from seven regions including *rbcL*, *rps4*, the 5′ *trnK* intron, *matK*, the 3′ *trnK* intron, *trnL-F*, and ITS. Bayesian posterior probabilities (BI), maximum likelihood bootstrap probabilities (ML), and maximum parsimony bootstrap probabilities (MP) are shown on each branch as (BI/ML/MP). Support values <50% are shown as hyphens (-). Scale bar indicates a branch length corresponding to 0.1 substitutions per site.

Figure 2 shows a majority rule consensus tree generated by BI analysis. The major five clades recognized in the analyses are indicated with Roman numerals (I–V). These clades were supported by high statistical values. Clade I contained *L. bowringii* Mitt. and *L. sumatranum* (Brid.) Hampe. ex M.Fleisch., and clade II contained *L. albidum* (P.Beauv.) Lindb., *L. glaucum*, and *L. juniperoideum*. Clades I and II were supported by high statistical support (Bayesian posterior probabilities/ML bootstrap/MP bootstrap = 1.00/100/100). Clade III contained only one species, *L. chlorophyllosum* Müll.Hal., from the Philippines and Indonesia. Clade IV contained *L. candidum* (Brid. ex Beauv.) and *L. aduncum* Dozy & Molk.. All three species contained in Clades III and IV are distributed in southeastern Asia and the south Pacific region. Clade V contained six species: *L. boninense* restricted to the Bonin Islands, *L. javense*, *L. scabrum*, *L. scaberulum,* and *L. pachyphyllum* from Oahu Island, and *L. seemannii* from Maui Island.

*Leucobryum boninense* from various islands in the Bonin Islands made a clade with strong statistical support (Bayesian posterior probabilities/ML bootstrap/MP bootstrap = 1.00/99/99), and was closely related to *L. scabrum* from Japan, Taiwan, and Hong Kong, and *L. javense* from Japan. Among the *L. boninense* samples, those from the Ogasawara Islands (Chichijima Island, Hahajima Island, and Anijima Island) and Kita-iwo Island showed a 1-bp difference in the *rbcL*, 1-bp deletion in the 5′ *trnK* intron, and 10-bp deletion in the ITS. The sequences of the *rps4*, *matK*, 3′ *trnK* intron and *trnL*-*F* were the same between them.

Three species, *L. scabrum*, *L. scaberulum*, and *L. javense* showed similar sequences to that of *L. boninense*. Our phylogenetic results showed that the plant samples of *L. scabrum* and *L. scaberulum* were monophylic, in contrast that those of *L. javense* were polyphyletic. *Leucobryum scaberulum* contained two different groups: the Ryukyus group consisting of plant materials from the Ryukyus and Taiwan and the China group consisting of those from Hong Kong and Guangdong. *Leucobryum javense* was divided into four clades, samples #1 and 2 from Japan were closely related to *L. boninense* and *L. scabrum*, sample #4 from Malaysia was sister to the Hawaiian endemic species, *L. pachyphyllum* and *L. seemannii*, samples #3, 5, and 6 were sister to *L. scaberulum*, and sample #7 from Malaysia formed an independent clade.

## Discussion

### Origin of *Leucobryum boninense*, endemic to the Bonin Islands, Japan

In this study, the endemic species *L. boninense* formed a robust clade with five related species including *L. scabrum*, *L. javense*, *L. scaberulum*, *L. pachyphyllum*, and *L. seemannii*, as suggested by Oguri et al. (2003, 2008) (Fig. 2; clade V), and was closely related to *L. scabrum* from Japan, Taiwan, and Hong Kong and *L. javense* from Japan. No differences in the *rps4* sequences were observed between the *L. boninense* samples and those of *L. scabrum*, in contrast, only 1-bp difference was observed in the *rps4* sequences between the *L. boninense* samples and those of *L. javense* from Japan. In the *rbcL* sequences, 1-bp difference was observed between the *L. boninense* samples from the Ogasawara Islands (Chichijima Island, Hahajima Island, and Anijima Island) and those of *L. boninense* from Kita-iwo Island, as well as between those of *L. boninense* from the Ogasawara Islands and those of *L. scabrum*. *Leucobryum boninense* samples from the Ogasawara Islands and *L. javense* from Japan had the same *rbcL* sequences. In morphological characters, Yamaguchi (1993) mentioned that *L. boninense* is morphologically similar in the absence of a central strand in the stem and perichaetia terminal on short lateral branches to *L. scabrum* and *L. javense*. However, this species is clearly distinguishable from *L. scabrum* based on leaves being papillose-prorate on the abaxial surface, and is also clearly distinguishable from *L. javense* based on its small plant size (Yamaguchi 1993). Therefore, this molecular phylogenetic result suggests that *L. boninense*, which is restricted to the Bonin Islands, originated from Japan, Taiwan, or Hong Kong. The bryophyte flora of the Bonin Islands is generally regarded as similar to that of East and Southeast Asia (Iwatsuki 1985). However, this is still the first demonstration that molecular phylogenetic data directly support an East Asian origin of a moss species endemic to the Bonin Islands.

### Origin of the Hawaiian endemic species of *Leucobryum*

In the case of Hawaiian mosses, their geographical origins remain unclear, although it is known that that Hawaiian moss flora, especially of cosmopolitan taxa, shows almost no connection with those of the American continents (Bartram 1933). *Leucobryum pachyphyllum* and *L. seemannii* are endemic to the Hawaii, and the two species are morphologically characterized by medium-sized plants and abaxially rough leaves (Bartram 1933; Staples et al. 2004). Our phylogenetic tree showed that the two species formed a monophyletic group, and were closely related to *L. javense* from Malaysia (Fig. 2). *Leucobryum albidum*, which is restricted in North America, formed a clade with *L. glaucum* from Japan and *L. juniperoideum* from Japan, and is genetically distinct from the Hawaiian *Leucobryum* (Fig. 2; clade II). This species is clearly distinguished from the Hawaiian endemic species by smooth abaxial leaf surface and terminal perichaetia on stems (Bartram 1933). Molecular phylogenetic results suggested that the two Hawaiian endemic species may be originated from a southeastern Asia, not from the America.

### Utility of the chloroplast *matK* gene for resolving phylogenetic relationships among *Leucobryum* species

Bryophyte phylogeny and biogeography have been studied using nucleotide sequence information of nuclear and plastid DNAs such as those of nuclear ITS regions, chloroplast *rbcL*, *rps4*, *trnG* and *trnL*-*F*, for resolving origin and species delimitation (e.g. Huttunen et al. 2008; Oguri et al. 2008; Shaw et al. 2008; Preußing et al. 2010; Villarreal et al. 2010). However, phylogenetic analyses using chloroplast *matK* have not been well performed yet in bryophytes, although this gene is a powerful source for angiosperm phylogenetic analyses (Rev. Müller et al. 2006). A molecular phylogenetic study of *Asterella* (Aytoniaceae, Marchantiopsida), inferred from partial *matK* sequences (aligned length = 759 bp) by Long et al. (2000), is the only study to date. Their phylogenetic analysis strongly supported monophyly of Aytoniaceae; therefore, they concluded that the *matK* region is a useful source of phylogenetic signals in *Asterrella* and related marchantioid liverworts. In the present study, we compared useful sequence information among each sequence data for 50 samples containing 15 species of *Leucobryum* (Table 3). A percentage of parsimony-informative characters in the *trnK* intron (5.8%) was significantly higher than other chloroplast DNA regions, *rbcL* and *rps4*, although its percentage in the ITS (25.6%) was the highest among the seven regions. Maximum parsimony trees based on the *trnK* intron sequence data (CI = 0.824, RI = 0.916) were relatively robust than those based on the *rbcL* (CI = 0.783, RI = 0.885), ITS (CI = 0.778, RI = 0.894), and the combined seven sequence data (CI = 0.768, RI = 0.862). Therefore, the sequence data of *trnK* intron region including *matK* provided more informative signals for phylogenetic reconstruction among *Leucobryum* species.

In the present study, we also sequenced the chloroplast *trnK* intron region including *matK* of six moss species from various taxonomic groups (Appendix S1). Among these six moss species, *Brothera leana* and *Dicranodontium denudatum* were mostly closely related to *Leucobryum* species, whereas the remaining four species had largely different *rbcL* sequences from *Leucobryum* species, according to the results of a previous molecular phylogenetic study by Tsubota et al. (2004). Therefore, six primers (four primers of the six were newly designed in the present study, Table 2) for the *trnK* intron and *matK* are expected to be useful for molecular phylogenetic analyses in various moss taxa.

## Supporting Information

**Appendix S1.** Six moss species analyzed in this study, their voucher information, and GenBank accession numbers of the DNA sequences.
