# Supplementary material for: Geographical origin of Leucobryum boninense Sull. & Lesq. (Leucobryaceae, Musci) endemic to the Bonin Islands, Japan
Source: Ecol Evol. 2013 Feb 15;3(4):753–62. doi: 10.1002/ece3.492 (PMC3631391; doi:10.1002/ece3.492)
Supplement: Supplementary file 1 [file ece30003-0753-SD1.doc]

Appendix S1 List of six moss species, voucher specimen, origin of sample and accession numbers.

| Taxon | Voucher specimen | Origin of sample | *trnK* intron |
| --- | --- | --- | --- |
| Tetraphidaceae |  |  |  |
| *Tetraphis pellucida* Hedw. | MAK B118667 | Japan. Nagano-ken | AB742509 |
| Dicranaceae |  |  |  |
| *Brothera leana* (Sull.) Müll.Hal. | HIRO 120176 | Taiwan. Taichung County | AB742510 |
| *Dicranodontium denudatum* (Brid.) E.G.Britt. ex Williams | HIRO 138866 | Japan. Yakushima Isl. | AB742511 |
| Hylocomiaceae |  |  |  |
| *Rhytidium rugosum* (Hedw.) Kindlb. | MAK B118582 | Japan. Nagano-ken | AB742512 |
| Hypnaceae |  |  |  |
| *Hypnum plumaeforme* Wilson | MAK B119226 | Japan. Tokyo | AB742513 |
| *Isopterygium propaguliferum* Toyama | MAK B119183 | Japan. Wakayama-ken | AB742514 |
